# Patent Foramen Ovale Closure for Splenic Infarction: An Unusual Presentation and an Unusual Indication

**DOI:** 10.1155/2020/9802908

**Published:** 2020-03-19

**Authors:** Edgar Stroppa Lamas, Alan Vinicius Gamero Osti

**Affiliations:** ^1^Department of Interventional Cardiology, Intercor, Hospital 13 de Maio, Sorriso, Brazil; ^2^Department of Interventional Cardiology, Intercor, Hospital Santo Antonio, Sinop, Brazil

## Abstract

**Background:**

Splenic infarction is a rare clinical condition. It is generally attributed to hematologic, vascular, cardioembolic, and infectious diseases or trauma. *Case Presentation*. We describe a rare case in an otherwise asymptomatic 41-year-old overweight woman with acute abdominal pain. Imaging work-up revealed splenic infarction. Common etiologies were excluded. A transesophageal echocardiography (TEE) revealed a patent foramen ovale (PFO). The patient was sent to closure with good outcome.

**Conclusion:**

Paradoxical embolism due to PFO can be a cause of splenic infarction, and its investigation and subsequent closure may be considered when there are no other causative disorders.

## 1. Introduction

Splenic infarction is a rare cause of acute abdominal pain which can mimic several other conditions. It is often the result of infiltrative hematologic disorders [1, 2]. Other conditions like hypercoagulable states, infective endocarditis, cardioembolic events, and more rarely, infectious mononucleosis may be associated [3].

Patent foramen ovale is encountered in about 25% of unselected patients [4]. Recent debates focus on its closure in cryptogenic stroke in young patients. Some reports describe other probably paradoxical cardioembolic events (renal, splenic) that may be benefited by patent foramen ovale closure [5, 6].

In this report, we present a rare cause of abdominal pain due to splenic infarction in which the etiology investigation points to paradoxical embolic infarction due to patent foramen ovale. Percutaneous closure was successfully done with good midterm outcome.

## 2. Case Presentation

A 41-year-old woman presented to the emergency department with acute epigastric pain radiating to the back associated with nausea. There was no history of diarrhea, fever, or trauma. There was no response to analgesics. Her medical history was unremarkable except class I obesity. On admission, the patient was apyrexial (36.7°C), eupneic at rest (respiratory rate of 14/min), and with a blood pressure of 130/80 mmHg and a pulse rate of 102 beats per minute. Cardiopulmonary examination was normal. There was localized left-sided and epigastric tenderness and no peritonism. Bowel sounds were normal.

An electrocardiogram (ECG) showed sinus tachycardia and no other abnormalities. Laboratory tests showed WCC 9.4 × 109/L, Hb 15 g/L, and CRP 24 mg/L. Serum levels of electrolytes, bilirubin, alkaline phosphatase, amilase, and creatinine were normal.

**Figure 1:**
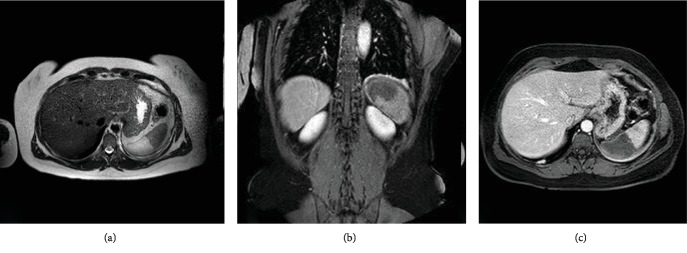
Abdominal MRI. Axial T2-weighted image (a) showing a high-intensity signal in the upper splenic portion. Coronal (b) and axial (c) T1-weighted fat suppressed postcontrast image revealing a hypovascularized area in the corresponding area.

Abdominal ultrasound did not show any abnormalities. Abdominal magnetic resonance imaging (MRI) was performed and revealed a heterogeneous signal on a splenic topographic, low-intensity, and hypovascularized area with a cuneiform shape on its upper portion (Figure 1).

The patient was tested for the inherited thrombophilic factors including protein C, protein S, or antithrombin deficiency; hyperhomocysteinemia; lupus anticoagulant and anticardiolipin antibodies; activated protein C resistance; and factor V Leiden mutation and prothrombin gene mutations. All tests were normal. A venous ultrasound of the lower limbs excluded deep venous thrombosis.

Therapeutic anticoagulation with enoxaparin (1 mg per kilogram subcutaneous) was started which provided a remarkable relief from the abdominal pain. Transthoracic echocardiogram was performed and revealed good biventricular function and no evidence of thrombus or valvular disease. The patient was then discharged asymptomatic with oral anticoagulation (rivaroxaban) prescribed.

On ambulatory follow-up one week later, the patient was on good evolution. Computed tomography angiography of the abdomen did not reveal any atherosclerotic patterns. Ambulatory monitoring with 24-hour Holter did not show paroxysmal atrial fibrillation or other significant supraventricular or ventricular arrhythmias.

**Figure 2:**
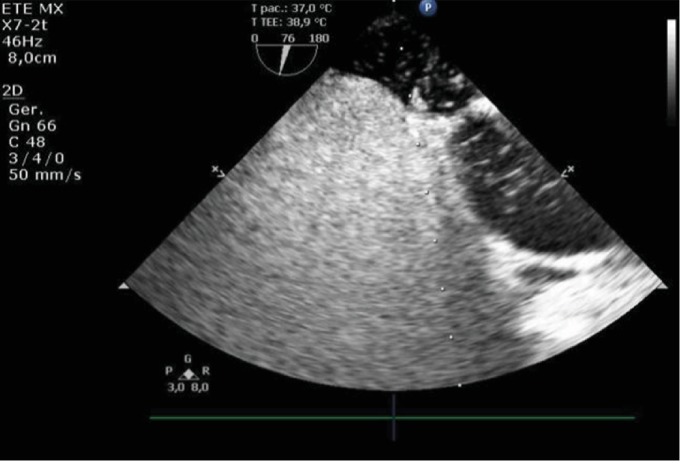
Transesophageal echocardiogram. Image revealing patent foramen ovale. After injection of agitated saline, a right-to-left shunt was observed.

A transesophageal echocardiogram (TEE) was done and revealed a patent foramen ovale with right-to-left shunt after agitated saline contrast injection during a Valsalva maneuver (Figure 2).

**Figure 3:**
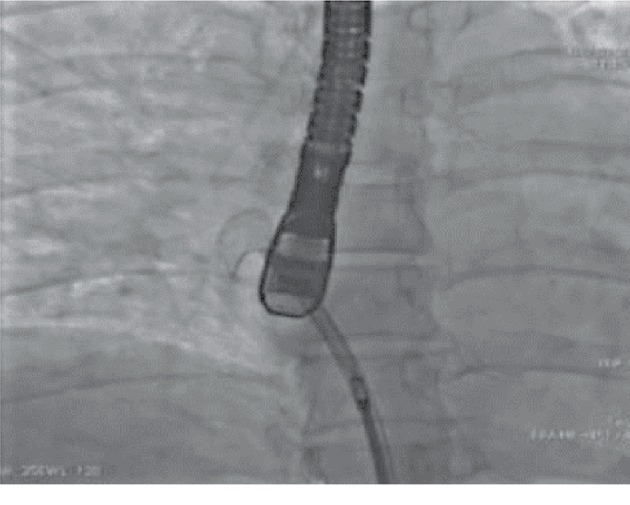
Percutaneous PFO closure with a Figulla Flex II PFO occluder. Right side portion of the device opened in the right atrium.

**Figure 4:**
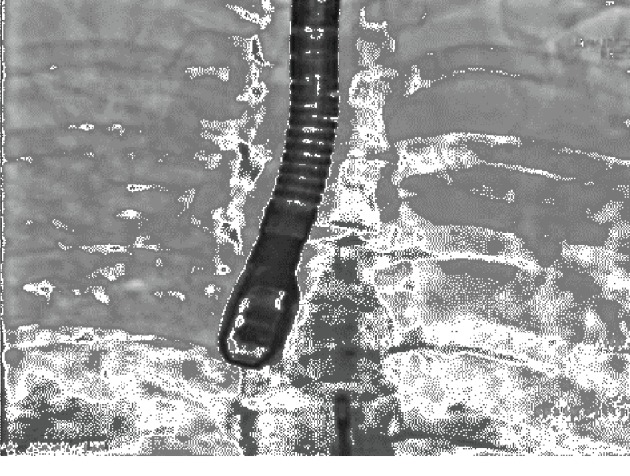
Percutaneous PFO closure with a Figulla Flex II PFO occluder. Left side portion of the device opened in the left right atrium.

Based on these findings, the patient underwent percutaneous closure of the patent foramen ovale with insertion of a 23/25 mm self-expandable Figulla Flex II PFO occluder (Occlutech, Helsingborg, Sweden). The device was inserted using the right femoral approach. The procedure was done with TEE guiding. Immediate angiography and echocardiographic result was excellent (Figures 3 and 4).

Acetylsalicylic acid 100 mg/d and clopidogrel 75 mg/d were prescribed for three months, and thereafter, aspirin was continued alone following implantation. Rivaroxaban was suspended after the procedure.

Clinical and echocardiographic follow-ups were performed after one and six months and revealed good echocardiographic results and no clinical events or abnormalities.

## 3. Discussion

There is recent intense debate on the management of embolic cryptogenic stroke in the presence of patent foramen ovale. In specific subjects, patent foramen ovale closure may be considered over medical treatment [7]. Despite the low incidence, other potential sources of embolic damage should be remembered leading to the investigation of this etiology and consideration for patent foramen ovale closure.

Splenic infarction is a rare and difficult condition to recognize. Hematologic disorders especially Sickle cell disease and thrombophilic conditions are usually considered in this context. Like our case, after full investigation, particularly in a young patient, patent foramen ovale diagnostic work-up and its respective closure may be considered.

## Abbreviations

PFO: Patent foramen ovale
TEE: Transesophageal echocardiography
MRI: Magnetic resonance image.
